# Assessing the accuracy and usefulness of YouTube videos on small incision lenticule extraction (SMILE) surgery for patients and healthcare professionals

**DOI:** 10.1007/s10103-025-04299-w

**Published:** 2025-01-25

**Authors:** Sibel Ahmet, Ali Safa Balci, Burcu Kemer Atik, Hüsna Topçu, Ahmet Kırgız

**Affiliations:** Department of Ophthalmology, University of Health Sciences, Beyoglu Eye Training and Research Hospital, Istanbul, 34420 Turkey

**Keywords:** Refractive surgery, Small incision lenticule extraction, Video, YouTube

## Abstract

To evaluate the quality, usefulness, and reliability of videos about Small Incision Lenticule Extraction (SMILE) surgery on the YouTube platform. On January 19, 2022, a search was performed on YouTube (www.youtube.com) with the keywords ‘small incision lenticule extraction’, ‘SMILE refractive surgery’, and ‘ReLex SMILE Surgery’. The default search option was “sort videos by relevance”. The duration of videos (seconds), the number of views, the source of videos (surgeons/medical organizations-health channels/patients-others), the number of subscribers, the number of likes and dislikes, like ratio (like × 100/[like + dislike]), the number of comments, the time since upload date (days), the video content (surgery/theoretical information), the content of surgical videos (real surgery/animation), mode of expression (verbal narration/subtitle), and the presence of a conflict of interest (yes/no) were recorded. The videos were blindly evaluated by two refractive surgeons (SA and AK) using DISCERN, the Journal of the American Medical Association (JAMA), and the Global Quality Score (GQS). A total of 101 videos were analyzed, 38 (37.6%) of which were uploaded by surgeons. The mean DISCERN, JAMA, and GQS were 37.73 ± 7.49; 1.90 ± 0.57; and 2.20 ± 0.73, respectively. The JAMA score, GQS, and video duration were all significantly correlated with the DISCERN score. The GQS was significantly correlated with all parameters except the JAMA score. Overall, SMILE surgery videos on the YouTube platform may provide cursory information to non-ophthalmologists; however, when the videos are evaluated using tools such as DISCERN, JAMA, and GQS, they are of low quality in terms of refractive surgery education for ophthalmologists. Experts should evaluate and review content uploaded to websites such as YouTube.

## Introduction

The internet age has accelerated our ability to access information. People can now find a wide variety of information on health-related topics online. This makes it very important to verify the accuracy and authenticity of online health-related information. YouTube is one of the most visited sites in the world, with more than one billion hours of video watched every day and billions of views, offering free video sharing [1]. It is also widely used as a consultant by doctors, medical students, and other health professionals [2–5].

The flapless approach known as Small Incision Lenticule Extraction (SMILE), introduced by Sekundo et al. in 2011, is a relatively new refractive surgery procedure used to correct myopia and myopic astigmatism, compared to photorefractive keratectomy (PRK) and laser-assisted in situ keratomileusis (LASIK) [6, 7]. The widespread use of SMILE in clinical practice is increasing its popularity among physicians and patients because it is a minimally invasive surgery with less impact on corneal biomechanics and subbasal nerve plexus [8]. Due to their limited training in SMILE surgery, ophthalmologists often search YouTube to learn more about the procedure. Patients considering refractive surgery may also turn to YouTube to learn about the procedure.

In addition to studies examining YouTube videos on refractive surgery, to our knowledge, our study is the first study to examine the quality of YouTube videos on SMILE surgery.

## Materials and methods

Institutional review board approval was not required for this retrospective, cross-sectional, record-based study. On 19 January 2022, a search was performed on YouTube (www.youtube.com) with the keywords ‘small incision lenticule extraction’, ‘SMILE refractive surgery’ and ‘ReLex SMILE Surgery’. The search was performed in the incognito tab of the browser without logging in and by clearing the search history. “Sort videos by relevance” was the default search option. The first 75 videos from the searches for each of the 3 keywords were included. A total of 225 videos were watched. Only videos in English were included. Videos in other languages, duplicate videos, videos related to PRK or LASIK, videos not related to refractive surgery, and videos shorter than 60 s were excluded from this study.

The duration of videos (seconds), the number of views, the source of videos (surgeons/ medical organizations-health channels/ patients-others), the number of subscribers, the number of likes and dislikes, like ratio (like × 100/[like + dislike]), the number of comments, time since upload date (days), the video content (surgery/theoretical information), the content of surgical videos (real surgery/animation), mode of expression (verbal narration/subtitle), presence of conflict of interest (yes/no) were recorded.

Two refractive surgeons viewed and analyzed the videos (SA, AK). The DISCERN questionnaire score, JAMA benchmarking criteria, and Global Quality Score (GQS) were used to assess the reliability and educational aspects of the videos. The DISCERN score system consists of three sections and sixteen questions. Each question is given a score between 1 and 5 (Table 1). If the answer to the question is a definite ‘yes’, a score of 5 should be given. If the criteria are partially met, a score of (2–4) should be given. If the answer to the question is a definite ‘no’, 1 point should be awarded. The objectiveness and exhaustibility of medical knowledge, particularly regarding therapy, are carefully evaluated by this rating system [9]. The first section assesses the reliability of the source, in this case, an online video, with 8 questions. In the second section, treatment-related information is reviewed with 7 questions [10]. The last section (question 16) consists of the overall quality assessment [11, 12]. The Journal of the American Medical Association (JAMA) scoring is a method used to assess the quality of data on health-related websites (Table 1). It consists of four criteria (authorship, attribution, disclosure, and currency) with a maximum score of four points and a maximum score of one point per criterion [13].

The GQS system is a scoring system used to evaluate a video for its informative aspects for patients (Table 1). The GQS method provides users with a five-point Likert scale to rate the overall quality of a video’s content [14]. A score of one point represents the lowest quality, and a score of five points represents the highest quality. The GQS system similarly mimics the flow and availability of the data displayed in the online video.

The SPSS version 26.0 was used for statistical analysis (SPSS Inc. Chicago, IL). The Kolmogorov-Smirnov test was used to determine the distribution and normality of the data. Mean and standard deviation (SD) were used to reveal continuous variables with a normal distribution. Continuous variables that do not have normal distribution are presented with median and range (R). Frequency and percentage were given for categorical data. For each video, the answers to the 16 questions (1–5) of the DISCERN score and the answers to the 4 questions (0–1) of the JAMA score were numerically summed separately. Statistics were calculated by averaging the total DISCERN and total JAMA scores separately for 101 videos. Then, the correlation between total DISCERN and total JAMA and GQS was evaluated by the Pearson correlation test. The chi-square test, one-way ANOVA (with Tukey post hoc test), and Kruskal-Wallis tests were used to compare parameters between groups when appropriate. A ‘P’ value < 0.05 was considered significant.

**Table 1:** DISCERN, JAMA and GQS scoring systems criteria

Discern							
Question Number	What is investigated?	Question Rating					
**Section 1**	**Is the publication reliable?**	**No**	**Partially**				**Yes**
1	Are the aims clear?	1	2	3		4	5
2	Does it achieve its aims?	1	2	3		4	5
3	Is it relevant?	1	2	3		4	5
4	Is it clear what sources of information were used to compile the publication (other than the author or producer)?	1	2	3		4	5
5	Is it clear when the information used or reported in the publication was produced?	1	2	3		4	5
6	Is it balanced and unbiased?	1	2	3		4	5
7	Does it provide details of additional sources of support and information?	1	2	3		4	5
8	Does it refer to areas of uncertainty?	1	2	3		4	5
**Section 2**	**How good is the quality of information regarding treatment choices?**						
9	Does it describe how each treatment works?	1	2	3		4	5
10	Does it describe the benefits of each treatment?	1	2	3		4	5
11	Does it describe the risks of each treatment?	1	2	3		4	5
12	Does it describe what would happen if no treatment is used?	1	2	3		4	5
13	Does it describe how the treatment choices affect overall quality of life?	1	2	3		4	5
14	Is it clear that there may be more than 1 possible treatment choice?	1	2	3		4	5
15	Does it provide support for shared decision making?	1	2	3		4	5
**Section 3**	**Overall rating of the publication**						
16	Based on the answers to all of these questions, rate the overall quality of the publication as a source of information about treatment choices	1	2	3		4	5
***The Journal of the American Medical Association Score***		**Yes**			**No**		
Authorship	Authors and contributors, their affiliations, and relevant credentials should be provided	1			0		
Attribution	References and sources for all content should be listed clearly, and all relevant copyright information should be noted	1			0		
Disclosure	Website “ownership” should be prominently and fully disclosed, as should any sponsorship, advertising, underwriting, commercial funding arrangements or support, or potential conflicts of interest	1			0		
Currency	Dates when content was posted and updated should be indicated	1			0		
***Global Quality Score***		**Question Rating**					
Poor quality, very unlikely to be of any use to patients		1					
Poor quality but some information present, of very limited use to patients		2					
Suboptimal flow, some information covered but important topics missing, somewhat useful to patients		3					
Good quality and flow, most important topics covered, useful to patients		4					
Excellent quality and flow, highly useful to patients		5					

JAMA: The Journal of the American Medical Association

GQS: Global Quality Score

## Results

One hundred and one of 225 videos were included in the study. Of these videos, 38 (37.6%) were uploaded by surgeons, 50 (49.5%) by medical organizations and health channels, and 13 (12.8%) by patients and others. Seventy-five (74.3%) of these videos describe surgical technique, 7 (6.9%) clinical examination/theoretical information, and 19 (18.8%) both. Seventy-six (75.2%) of the videos were live surgery and 13 (12.9%) were animations. Seven (6.9%) videos included both live surgery and animation. Five (5%) of the videos did not include live surgery or animation but were in the form of verbal narration. There was audio narration in 28 (27.7%) videos and subtitled narration in 18 (17.8%) videos. Both types of narration were present in 9 (8.9%) videos. There was no audio or subtitled narration in 46 (45.5%) of the videos. There was a conflict of interest in 27 (26.7%) of the videos. The mean DISCERN, JAMA and GQS were 37.73 ± 7.49; 1.90 ± 0.57; 2.20 ± 0.73, respectively. Other findings are listed in Table 2.

**Table 2:** Descriptive statistics of YouTube videos. (*n* = 101)

Parameters	Median	Range	Min-Max	Mean±Standard deviation
Video duration	220	837	63–900	259.76 ±177.07
Number of views	292	2,275,767	4-2275771	41746.98 ± 265172.00
Number of likes	3	12,000	0-12000	196.68 ± 1295.04
Number of dislikes	0	779	0-779	16.19 ± 103.93
Number of subscribers	69	1,940,000	0-1940000	24017.62 ± 193305.94
Number of comments	0	1603	0-1603	26.30 ± 167.33
Like Ratio	100	37.50	62.50–100	97.35 ± 6.54
DISCERN SCORE	36	46	29–75	37.73 ± 7.49
JAMA SCORE	2	4	0–4	1.90 ± 0.57
GQS	2	4	1–5	2.20 ± 0.73

JAMA: The Journal of the American Medical Association

GQS: Global Quality Score

Video sources were divided into three groups and mean scores were analyzed. For videos uploaded by surgeons (*n* = 38, 37.6%), the mean DISCERN score was 35.57 ± 5.68 and the mean JAMA score was 1.97 ± 0.28. For videos uploaded by medical organizations-health channels (*n* = 50, 49.5%), the mean DISCERN score was 40.12 ± 8.61 and the mean JAMA score was 1.94 ± 0.71. For videos uploaded by patients-others (*n* = 13, 1.3%), the mean DISCERN score was 34.84 ± 4.20 and the mean JAMA score was 1.53 0.51. Other findings of this comparison are listed in Table 3.

**Table 3:** Comparison of video parameters according to the different upload sources

Parameters (Mean±Standard deviation)	Surgeons (*n* = 38, 37.6%)	Medical organizations-Health channels (*n* = 50, 49.5%)	Patients-Others (*n* = 13, 12.9%)	*P* value
Video duration	230.89 ± 161.90	247.47 ± 154.94	403.42 ± 248.29	**0.009** ^**a**^
Number of views	2890.84 ± 7119.161	52472.67 ± 317999.51	119207.25 ± 408802.98	0.386^a^
Number of likes	16.26 ± 46.64	274.47 ± 1677.19	437.42 ± 1499.83	0.517^a^
Number of dislikes	0.21 ± 0.93	18.02 ± 108.90	59.00 ± 203.43	0.231^a^
Number of subscribers	1334.87 ± 5853.77	7228.35 ± 22337.36	167200.75 ± 558445.04	**0.022** ^**a**^
Number of comments	1.79 ± 7.47	40.41 ± 225.47	43.92 ± 141.28	0.524^a^
Like Ratio	99.33 ± 2.57	96.57 ± 6.5	93.82 ± 13.32	0.054^a^
DISCERN SCORE	35.57 ± 5.68	40.12 ± 8.61	34.84 ± 4.20	**0.005** ^**a**^
JAMA SCORE	1.97 ± 0.28	1.94 ± 0.71	1.53 ± 0.51	**0.048** ^**a**^
GQS	2.08 ± 0.53	2.31 ± 0.83	2.08 ± 0.79	0.282^b^

JAMA: The Journal of the American Medical Association

GQS: Global Quality Score

Bold values indicate statistical significance

a: One-way ANOVA test

b: Chi-Square test

Mean DISCERN and JAMA scores differed significantly between videos uploaded by the three groups (*p=0.005, p=0.048, respectively* ).

Mean DISCERN scores were significantly different between videos uploaded by surgeons and videos uploaded by healthcare organizations-health channels (*p* < 0.05, post hoc Tukey).

Mean JAMA scores were statistically significantly different between videos uploaded by surgeons and videos uploaded by patients-others (*p* < 0.05, post hoc Tukey).

When the GQS score of the videos was evaluated, there was no statistically significant difference between the 3 groups (*p* > 0.05, chi-square). In addition, the mean number of views of videos in the second and third groups exceeded that of the videos uploaded by surgeons, but not statistically significant (*p* > 0.05).

Table 4 presents the correlations between DISCERN, JAMA, GQS, number of views, number of subscribers, number of comments, number of likes, number of dislikes and video duration. A significant relationship emerged between DISCERN score and GQS, JAMA score and video duration. However, JAMA score was not correlated with any other parameter except DISCERN score. Furthermore, GQS was significantly associated with all parameters except the JAMA score. Finally, videos with more views tended to receive significantly more likes, dislikes, and comments.

**Table 4:** Correlation between DISCERN, JAMA, GQS, and video parameters

	DISCERN	JAMA	GQS	Number of views	Number of subscribers	Number of comments	Number of likes	Number of dislikes	Video duration
DISCERN		*r* = 0.27* *p*** < 0.05**	*r* = 0.67** *p*** < 0.001**	*r* = 0.14 *p* > 0.05	*r* = 0.06 *p* > 0.05	*r* = 0.13 *p* > 0.05	*r* = 0.14 *p* > 0.05	*r* = 0.13 *p* > 0.05	*r* = 0.28* *p*** < 0.05**
JAMA			*r* = 0.071 *p* > 0.05	*r* = 0.019 *p* > 0.05	*r*=-0.005 *p* > 0.05	*r*=-0.019 *p* > 0.05	*r* = 0.016 *p* > 0.05	*r* = 0.015 *p* > 0.05	*r* = 0.114 *p* > 0.05
GQS				*r* = 0.26* *p*** < 0.05**	*r* = 0.24* *p*** < 0.05**	*r* = 0.26* *p*** < 0.05**	*r* = 0.27* *p*** < 0.05**	*r* = 0.25* *p*** < 0.05**	*r* = 0.29* *p*** < 0.05**
Number of views					*r*=-0.001 *p* > 0.05	*r* = 0.96** *p*** < 0.001**	*r* = 0.98** *p*** < 0.001**	*r* = 0.98** *p*** < 0.001**	*r* = 0.20 *p* > 0.05
Number of subscribers						*r* = 0.004 *p* > 0.05	*r*=-0.001 *p* > 0.05	*r*=-0.001 *p* > 0.05	*r* = 0.35** *p*** < 0.001**
Number of comments							*r* = 0.98** *p*** < 0.001**	*r* = 0.89** *p*** < 0.001**	*r* = 0.14 *p* > 0.05
Number of likes								*r* = 0.94** *p*** < 0.001**	*r* = 0.17 *p* > 0.05
Number of dislikes									*r* = 0.22* *p*** < 0.05**
Video duration									

JAMA: The Journal of the American Medical Association

GQS: Global Quality Score

Bold values indicate statistical significance

Pearson correlation test. **Correlation is significant at the 0.01 level. *Correlation is significant at the 0.05 level

## Discussion

Today, people consult the internet for every topic they are curious about; health is one of these topics. Especially patients considering surgery satisfy their curiosity about the process on social media. Besides patients, surgeons also use these channels to learn different surgical techniques or to go over what they have learned [15, 16]. Among these channels, YouTube is one of the most well-known and popular platforms with more than 5 billion daily views [17]. The purpose of this study was to assess the effectiveness of the YouTube videos on SMILE surgery and their usefulness to both patients and surgeons.

Satisfying the curiosity of the patient and improving the practice of the surgeon depends on the quality of the content. In this study, we evaluated the content of SMILE surgical videos using DISCERN, JAMA, and GQS systems. The mean scores were 37.73 ± 7.49 for DISCERN, 1.90 ± 0.57 for JAMA, and 2.20 ± 0.73 for GQS. According to these findings, when patients and surgeons view YouTube videos to research SMILE surgery, they obtain a moderate level of information. However, the results of the correlation analysis suggest that if high-quality educational videos are desired, videos with a high number of likes, dislikes, and subscribers should be selected. It should not be inferred from this result that videos with a high number of dislikes are more educational. We think that videos with a high number of dislikes are also more likely to be watched and interacted with.

Since we interpreted the video parameters by categorizing them into three groups rather than as a total (Table 3), although the GQS of the videos uploaded by surgeons and non-surgeons were similar, the number of views was higher for videos uploaded by non-surgeons. In similar studies in the literature, this is interpreted as the videos uploaded by non-surgeons to patients are clearer, and therefore the number of views of these videos is higher [18]. Of course, these videos are not only watched by patients, surgeons who want to improve their experience also watch these videos, but their contribution to this rate is very low since their number is less than the number of patients.

YouTube videos should be clearer, avoid medical terms, and provide patients with more comprehensive information about the process to be more efficient for patients. Instead of advertising a specific surgical technique, patients should be reminded that each disease is managed individually, that there is more than one treatment option, but that it is right to choose the appropriate method with their doctor. While describing treatment methods, attention should be paid to details that will not increase the patient’s anxiety and improve cooperation and treatment outcome.

While there are authors who consider surgical education from YouTube videos inappropriate, there are also authors who recommend that experienced surgeons upload their videos to YouTube channels and share them for educational purposes to support this method and even strengthen this channel as a resource [17]. On the other hand, for surgeons who want to improve themselves on the YouTube platform, the content of the videos should be different from those intended for patients. By referring to medical sources, classifying cases according to their difficulty, differentiating standard surgery from advanced surgery, and describing the management of complications, experiences can be shared and contribute to the development of surgeons in different geographies. In this way, videos can be labeled ‘for patients’ and ‘for healthcare professionals’, making them more efficient for both groups.

Although numerous studies have recently been published on the quality and usefulness of YouTube videos as a source of medical information, fewer ophthalmology-related videos have been evaluated [3, 5, 17]. According to the study by Kalaycı et al., patients who watched keratoplasty videos on YouTube were able to access fair information about the treatment. They also found that videos uploaded by doctors had statistically higher evaluation scores, were more useful and trustworthy, and were watched more [19]. Refractive surgery videos on YouTube were evaluated by Kucuk et al. and they concluded that YouTube videos were inadequate for patient education. They also found that videos uploaded by doctors and videos uploaded by non-doctors were of similar usefulness, but videos uploaded by non-doctors were viewed more frequently [18]. In their study of YouTube videos on refractive eye surgery, Vought et al. observed that physician-authored videos exhibited greater transparency in their authorship and utilized graphics and figures more frequently than patient-authored videos, which predominantly addressed risks, benefits, and quality-of-life issues [20]. In our study, similar to the study by Kucuk et al., we found that the number of views of videos uploaded by surgeons was lower, but unlike this study, the videos provided moderate information to YouTube users according to DISCERN, JAMA, and GQS scores. The differences in these studies may be related to the possibility that the frequency of surgeries performed may be different. Keratoplasty surgery is less common than refractive surgery, whereas SMILE surgery is one of the most recent refractive surgery options.

One of the limitations of the study is that YouTube is a volatile platform. New videos are added every second and videos added in the past can be deleted. Therefore, the results may vary according to the search date. Secondly, the results may also vary according to the keyword used. Although we included the first 75 videos with 3 different keywords, we may not have included more efficient videos with different keywords. Third, not all videos were homogeneous in terms of narration; some had audio, some had subtitles, some had both or neither. This may have affected the number of likes and views. Despite these limitations, the strengths of our study are that the videos included in our study were evaluated by two different ophthalmologists who did not log in to YouTube with their own accounts and were not affected by cookies and search history. However, more video evaluations are needed in the future for more reliable results.

In conclusion, as with other topics, searches on YouTube about SMILE surgery are increasing day by day. From the patients’ point of view, better quality content should be created that not only satisfies the patient’s curiosity but also contributes to improving surgical treatment outcomes and increasing patient compliance with the surgery. From the point of view of healthcare professionals, it may be more instructive to share cases with different levels of difficulty with the restriction ‘for healthcare professionals’, rather than describing routine SMILE surgery in a similar way. Indeed, sharing complication management by experienced surgeons may improve surgical outcomes for many patients across large geographies.
